# Management von Komplikationen im oberen Gastrointestinaltrakt nach externen Primäreingriffen: Eine Fallserie über komplexe Verläufe und die Weiterbehandlung an einem High-Volume-Zentrum

**DOI:** 10.1007/s00104-026-02553-7

**Published:** 2026-08-10

**Authors:** Loreen Sophie Rudek, Naita M. Wirsik, Hans A. Schlößer, Christiane J. Bruns

**Affiliations:** Universität zu Köln, Medizinische Fakultät und Uniklinik Köln, Klinik und Poliklinik für Allgemein‑, Viszeral‑, Thorax- und Transplantationschirurgie, Köln, Deutschland

**Keywords:** Krankenhauszentralisierung, Mindestmengen, Patientensicherheit, Komplikative Ersteingriffe, Weiterbehandlung an Zentren, Hospital centralization, Minimum volume, Patient safety, Complications, Secondary treatment at centers

## Abstract

**Hintergrund:**

Die Krankenhauszentralisierung und die Implementierung von Mindestmengen sind zentrale Aspekte der gegenwärtigen Gesundheitspolitik, die die medizinische Versorgung beeinflussen und auch Auswirkungen auf die Versorgung im Fall von Komplikationen haben.

**Ziel der Arbeit (Fragestellung):**

Die vorliegende Fallstudie arbeitet auf, welche Ressourcen an einem tertiären Zentrum für die Behandlung von extern komplikativ verlaufenden Fällen erforderlich sind.

**Material und Methoden:**

In die prospektive Single-Center-Studie wurden Patient:innen (Pat.) eingeschlossen, die im Zeitraum vom 01.01.2023 bis zum 31.12.2024 extern eine komplikative Operation, Intervention oder Diagnostik im Bereich des oberen Gastrointestinaltraktes erhielten und anschließend in der Klinik und Poliklinik für Allgemein‑, Viszeral‑, Thorax- und Transplantationschirurgie der Uniklinik Köln weiterbehandelt wurden.

**Ergebnisse:**

In unserem Zentrum wurden 39 Pat. (männlich: 38,5 %, weiblich: 61,5 %; Durchschnittsalter: 68,1 Jahre) nach externen komplikativen Primäreingriffen behandelt. Die führenden Komplikationen waren Perforationen, Nahtinsuffizienzen sowie inkomplette Resektionen/Rekonstruktionen. Nach externem Auftreten einer Komplikation wurden 15 Pat. direkt verlegt. Bei 24 Pat. wurden extern interventionelle und/oder operative Revisionen durchgeführt. Zum Zeitpunkt der Übernahme waren 71,8 % der Pat. intensivpflichtig. Nach Zentrumsverlegung erfolgten kumulativ 115 Operationen und 243 Ösophagoduodenoskopien (ÖGDs) mit 171 endoskopischen Vakuumtherapie-(EndoVAC-)Wechseln. Im Verlauf konnten 25,6 % der Pat. heimatnah verleget und 61,5 % in die Häuslichkeit entlassen werden. Die Mortalität lag bei 12,8 %.

**Diskussion:**

Die Behandlung von Pat. mit extern komplikativ verlaufenden Primäreingriffen erfordert erhebliche personelle, logistische und medizinische Ressourcen – insbesondere für umfangreiche Diagnostik, komplexe operative Versorgung und intensivmedizinische Betreuung.

**Graphic abstract:**

**Figure:**
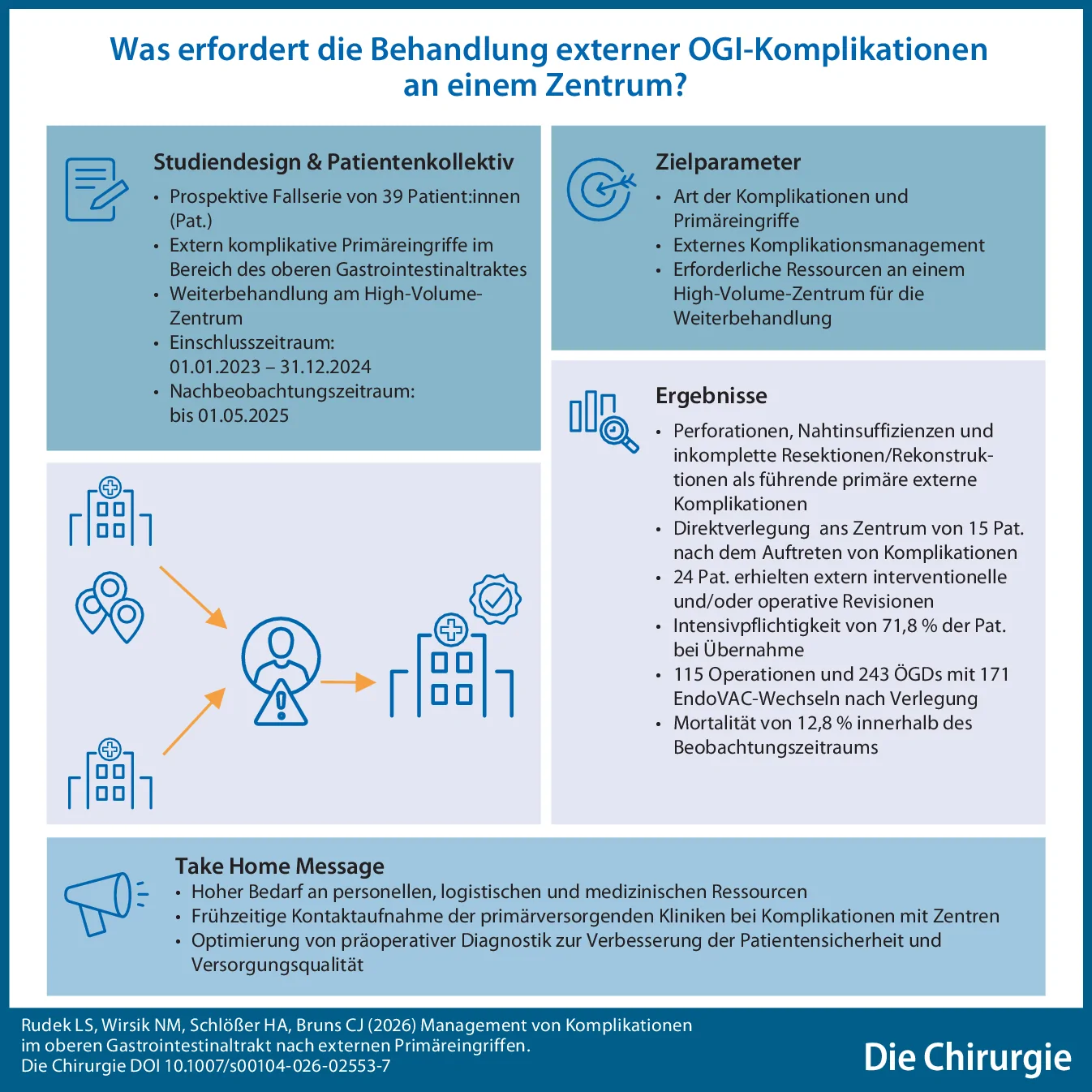

**Zusatzmaterial online:**

Die Online-Version dieses Beitrags (https://doi.org/10.1007/s00104-026-02553-7) enthält Tab. 5 mit der beispielhaften Fallserienaufarbeitung.

## Abstract (en)

**Background:**

The centralization of hospital services and the introduction of minimum volume requirements represent key pillars of modern health policy, affecting both the quality of care and the handling of clinical complications.

**Objective:**

This case study investigates the resources required at a tertiary or high-volume care center for the further treatment of patients with externally complicated primary procedures.

**Material and methods:**

The prospective single-center study included patients who underwent externally complicated surgery, intervention, or diagnostic procedure in the upper gastrointestinal tract between 1 January 2023 and 31 December 2024, and were subsequently treated at the Department of General, Visceral, Thoracic, and Transplantation Surgery of the University Hospital Cologne.

**Results:**

A total of 39 patients (male: 38.5%, female: 61.5%; average age: 68.1 years) were treated at our center after complicated primary procedures at external hospitals. The most common complications were perforation, suture insufficiency, and incomplete resection/reconstruction. Following occurrence of a complication 15 patients were transferred directly to the high-volume center. Interventional and/or surgical revisions were performed on 24 patients at external hospitals. At the time of transfer 71.8% of patients were in need of intensive care. A cumulative total of 115 surgical and 243 endoscopic procedures with 171 endoscopic vacuum (EndoVAC) changes were performed after transfer to the high-volume center. Over time, 25.6% of patients were readmitted to a local facility and 61.5% were discharged home. The mortality rate was 12.8 %.

**Conclusion:**

The treatment of patients with externally complicated primary procedures requires considerable personnel, logistical, and medical resources, especially for extensive diagnostics, complex surgical care, and intensive medical care.

Komplikationen nach Primäreingriffen im Bereich des oberen Gastrointestinaltraktes können durch patientenbezogene, technische oder strukturelle Faktoren entstehen und erfordern eine komplexe Diagnostik, interdisziplinäre Therapie und klinische Infrastruktur. Insbesondere bei komplexen Verläufen ist häufig ein spezialisiertes Management erforderlich. Die vorliegende Fallserie analysiert die Weiterbehandlung in einem universitären High-Volume-Zentrum von Patient:innen nach extern komplikativ verlaufenden Primäreingriffen im Bereich des oberen Gastrointestinaltraktes.

## Hintergrund und Fragestellung

Die Krankenhauszentralisierung und die Implementierung von Mindestmengen sind zentrale Aspekte der gegenwärtigen Gesundheitspolitik, die die medizinische Versorgung beeinflussen. Seit 2004 definiert der Gemeinsame Bundesausschuss (G-BA) Mindestmengen für planbare stationäre Leistungen als Instrument der Versorgungsqualität [1, 2]. Im Jahr 2021 erfolgte eine weitere wichtige Konkretisierung der Mindestmengenregelung (Mm-R) gemäß § 136b Absatz 1 Satz 1 Nummer 2 SGB V für nach § 108 SGB V zugelassene Krankenhäuser [3]. Bisweilen legte der G‑BA einen Katalog planbarer Leistungen dar, bei denen die Qualität des Behandlungsergebnisses von der Menge der erbrachten Leistungen abhängig ist, sowie die Festlegung von Mindestmengen für die entsprechenden Leistungen [4]. Trotz definierter Mindestmengen wurde ein Ausnahmetatbestand bei Nichterreichen einer Mindestmenge eingeräumt. So konnten Krankenhäuser gewisse Leistungen bei nachgewiesener hoher Qualität auch ohne Erreichen der vorgeschriebenen Mindestmengen erbringen. Mit der Anpassung der Mm‑R im Jahr 2021 wurde die Einräumung eines Ausnahmetatbestandes bei Nichterreichen der Mindestmenge gestrichen [3]. Folglich erlosch bei Erbringen einer entsprechenden Leistung und Nichterreichen der Mindestmenge der Anspruch auf Vergütung durch die gesetzliche Krankenkasse. Eine ausnahmsweise Leistungserbringung mit entsprechendem Vergütungsanspruch gilt so beispielsweise nur noch bei erstmaliger oder erneuter Erbringung einer entsprechenden Leistung [5].

### Mindestmengen im Bereich der Ösophaguschirurgie

Für komplexe Eingriffe am Organsystem Ösophagus für Erwachsene wurde mit der Änderung der Mm‑R vom 17.12.2020, welche am 01.01.2021 in Kraft getreten ist, eine jährliche Mindestmenge von 26 Eingriffen pro Standort eines Krankenhauses festgelegt [6]. Eine Übergangsregelung definierte für die Kalenderjahre 2021 und 2022 eine temporäre Mindestmenge von 10 Leistungen (komplexe Eingriffe am Organsystem Ösophagus für Erwachsene) pro Standort eines Krankenhauses. Mit dem Kalenderjahr 2023 endete die Übergangsregelung zur Erbringung von Mindestmengen.

Die Mindestmenge von 26 komplexen Eingriffen am Organsystem Ösophagus für Erwachsene ist auch Bestandteil der aktuellen Fassung der Mm‑R gemäß § 136b Absatz 1 Satz 1 Nummer 2 SGB V für nach § 108 SGB V zugelassene Krankenhäuser, welche mit Wirkung vom 01.01.2026 in Kraft getreten ist [7].

### Erfassung von Qualität in der Chirurgie

Das Institut für Qualität und Wirtschaftlichkeit im Gesundheitswesen (IQWiG) legte bereits 2020 einen positiven Zusammenhang zwischen einer Leistungsmenge und der Qualität von Behandlungsergebnissen am Beispiel von komplexen Operationen am Organsystem Ösophagus dar [8]. Im Zuge der laufenden Umstrukturierung der Krankenhauslandschaft ist davon auszugehen, dass die Implementierung von Mindestmengen sowie die Zentralisierung stationärer Leistungen langfristig auch Veränderungen in den Versorgungsstrukturen von Patient:innen im Fall von Komplikationen bewirken. Die vorliegende Fallserie legt exemplarisch für Eingriffe und Komplikationen im Bereich des oberen Gastrointestinaltraktes dar, welche Ressourcen an einem tertiären Zentrum/High-Volume-Zentrum für die Behandlung von extern komplikativ verlaufenden Fällen erforderlich sind.

## Studiendesign und Untersuchungsmethoden

Im Sinne einer prospektiven Single-Center-Studie wurden die Krankheitsverläufe von Patient:innen, welche extern komplikativ verlaufende Operationen oder Interventionen im Bereich des oberen Gastrointestinaltraktes erhielten und für die Weiterbehandlung im Zeitraum vom 01.01.2023 bis zum 31.12.2024 an die Universitätsklinik Köln verlegt wurden, als Fallstudie analysiert. Der Nachbeobachtungszeitraum erstreckte sich bis zum 01.05.2025.

Die Klinik und Poliklinik für Allgemein‑, Viszeral‑, Thorax- und Transplantationschirurgie (ehemals Klinik und Poliklinik für Allgemein‑, Viszeral‑, Tumor- und Transplantationschirurgie) des Universitätsklinikums Köln ist ein durch die Deutsche Gesellschaft für Allgemein- und Viszeralchirurgie zertifiziertes nationales Exzellenzzentrum für den oberen Gastrointestinaltrakt sowie ein durch die Deutsche Krebsgesellschaft zertifiziertes Speiseröhrenkrebszentrum. Als High-Volume-Zentrum für onkologische und funktionelle Operationen im Bereich des oberen Gastrointestinaltrakts liegen die Leistungszahlen über der geforderten Mindestmenge im Bereich der Ösophaguschirurgie (Ösophagektomien: in 2023 *n* = 211; in 2024 *n* = 196).

In die Fallstudie wurden Patient:innen mit einer der Primäroperation/-intervention zugrunde liegenden benignen und/oder malignen Grunderkrankung eingeschlossen. Des Weiteren wurden Patient:innen eingeschlossen, bei denen extern eine Diagnostik in einer Komplikation resultierte. Zu den erhobenen Falldaten zählen unter anderem Geschlecht, Alter, Grunderkrankung, Primäreingriff, Komplikation, externe und interne Revision(en), externe und interne interkurrente Diagnose(n), zuweisendes Krankenhaus, Art und Zeitpunkt der Zuweisung, Zuweisungsgrund, Dauer der Intensivpflichtigkeit, Beatmungsdauer, Hospitalisierungsdauer sowie Entlassung/Exitus letalis.

## Ergebnisse

### Extern komplikativ verlaufende Eingriffe

Im Beobachtungszeitraum wurde ein Kollektiv von 39 Patient:innen erfasst, von denen 15 männlich (38,5 %) und 24 weiblich (61,5 %) waren. Das durchschnittliche Alter der Patient:innen lag bei 68,1 Jahren (männlich: 65,7 Jahre [Range: 55 bis 80 Jahre], weiblich 69,5 Jahre [Range: 53 bis 90]). Von den insgesamt 39 Patient:innen wiesen 20 eine benigne und 19 eine maligne Grunderkrankung auf, aufgrund der die initiale Operation, Intervention oder Diagnostik im erstbehandelnden Krankenhaus erfolgte. Die Tab. 1 zeigt eine Gliederung der benignen und malignen Grunderkrankungen sowie Tab. 2 die Art der externen Primäreingriffe.

**Tab. 1:** Gliederung der benignen und malignen Grunderkrankungen der 39 Patient:innen, die bei extern komplikativ verlaufenden Eingriffen an der Uniklinik Köln weiterbehandelt wurden

Benigne Grunderkrankungen	Maligne Grunderkrankungen
Adipositas per magna	Adenokarzinom des gastroösophagealen Übergangs
Magenulkus	Adenokarzinom des Magens
Thoraxmagen	Plattenepithelkarzinom des Ösophagus
Hiatus‑/Zwerchfellhernie	Cricopharyngealer Tumor
Ösophagusstenose bei Ösophagitis	Lymphknotenrezidiv eines Karzinoids der Lunge
Zenker-Divertikel	–
Diskusprolaps der Halswirbelsäule	–
Kardiologische Grunderkrankungen	–

**Tab. 2:** Art der Primäreingriffe und Komplikationen der 39 Patient:innen, die bei extern komplikativ verlaufenden Eingriffen an der Uniklinik Köln weiterbehandelt wurden

Externe Primäreingriffe: Operation, Intervention, Diagnostik (Anzahl)	Externe Komplikationen (Anzahl)
Ösophagektomie mit Magenhochzug (11)	Perforation (23)
(Sub)totale Gastrektomie (6)	Anastomosen‑/Nahtinsuffizienz (10)
Fundoplikatio/Hiatoplastik (6)	Interponatischämie (1)
Divertikuloösophagostomie (2)	Postoperative Inkarzeration (1)
Bariatrische Operation (3)	(Broncho)ösophageale Fistel (3)
Diagnostische/therapeutische Endoskopie (5)	Chylothorax (2)
Videoassistierte Thoraxchirurgie (VATS) (1)	Blutung (2)
Thoraxdrainagenanlage (1)	Abdomen apertum (2)
Transösophageale Echokardiographie (TEE) (3)	Operationsabbruch (2)
Anteriore zervikale Diskektomie und Fusion (2)	Postoperative funktionelle Störung (1)
–	Verletzung umliegender Strukturen (3)

Die führende Komplikation bei Primäreingriffen bei benignen Grunderkrankungen waren Perforationen des Ösophagus oder Magens mit begleitender Sepsis auf dem Boden einer Mediastinitis oder Peritonitis (Tab. 2). Komplikationen der operativen Versorgung von Malignomen unterteilten sich unter anderem in Anastomoseninsuffizienzen, Perforationen und inkomplette Resektionen oder Rekonstruktionen im Sinne eines Operationsabbruches (Tab. 2).

Die Auswertungen (Tab. 3) zeigen Unterschiede zwischen den Zeitpunkten des Primäreingriffes, des Auftretens von Komplikationen und der Kontaktaufnahme mit der Uniklinik Köln durch die erstbehandelnden Krankenhäuser. Die Komplikationen traten bei allen Patient:innen während oder im direkten Zusammenhang mit dem Primäreingriff auf. Bei 37 Patient:innen handelte es sich um eine Direktverlegung aus den primär behandelnden Klinken. Bei 2 Patient:innen erfolgte die stationäre Aufnahme und Weiterbehandlung aus dem ambulanten Bereich. Die stationären Übernahmen erfolgten in allen Fällen unmittelbar nach der primären Kontaktaufnahme. In 69,2 % der Fälle erfolgte die Kontaktaufnahme und Übernahme der Patient:innen innerhalb der ersten 30 Tage nach Primäreingriff, in 30,8 % der Fälle nach mehr als 30 Tagen. Die späteste Übernahme ohne vorherige Ambulantisierung erfolgte 57 Tage nach dem komplikativen Ersteingriff. Bis zur Verlegung in das weiterbehandelnde Zentrum wurden bei 17 Patient:innen extern Revisionseingriffe durchgeführt. In 7 Fällen erfolgten extern 2 bis 6 Revisionsoperationen, bis eine Zentrumsverlegung stattfand. Weitere 18 Patient:innen erhielten extern eine Behandlung der Komplikationen im Sinne einer interventionellen endoskopischen Therapie (endoskopische Vakuumtherapie (EndoVAC), Stent, Clipping). Folglich wurden 9 Patient:innen extern mit einer Kombination aus Operation und interventioneller Endoskopie versorgt; 15 Patient:innen wurden direkt nach Eintreten der Komplikation an unser Zentrum verlegt.

**Tab. 3:** Kennzahlen der extern komplikativ verlaufenden Primärversorgung

Weitere Kennzahlen der externen Primärversorgung der 39 Patient:innen	
17 Patient:innen: extern operative Revision	(7 Patient:innen: 2 bis 6 Revisionsoperationen)
18 Patient:innen: extern endoskopische Revision	(EndoVAC, Stent, Clipping)
17 Patient:innen: Kombination aus externer operativer und interventioneller Revision	–
15 Patient:innen: Direktverlegungen ohne externe Revision	–
28 Patient:innen: intensivpflichtig bei Verlegung	–
10 Patient:innen: beatmungspflichtig bei Verlegung	–
37 Patient:innen: Verlegung ohne vorherige Ambulantisierung	(69,2 %: Verlegung innerhalb von 30 Tagen nach Auftreten der Komplikation, 30,8 %: Verlegung nach 30 Tagen nach Auftreten der Komplikation)

### Weiterbehandlung der Komplikationen am Zentrum

Bei Übernahme benötigten 71,8 % der Patient:innen eine intensivmedizinische Überwachung. Im weiteren stationären Aufenthalt in der Uniklinik Köln wurden 35 der 39 Patient:innen (89,7 %) intensivmedizinisch behandelt. Die Liegedauer auf der Intensivstation des weiterbehandelnden Zentrums lag bei 1 bis 110 Tagen (durchschnittlich 22 Tage) mit einer Beatmungsdauer von 1 bis 43 Tagen. In 2 Fällen war eine kardiopulmonal-unterstützende Therapie mit einer extrakorporalen Membranoxygenierung (ECMO) erforderlich. Insgesamt waren 25,6 % der Patient:innen zum Zeitpunkt der Übernahme beatmungspflichtig. Die totale Krankenhausliegedauer nach Zentrumsverlegung lag bei durchschnittlich 44 Tagen pro Patient:in (Range: 6 bis 131 Tage) (Tab. 4).

**Tab. 4:** Kennzahlen der Weiterbehandlung an der Klinik und Poliklinik für Allgemein‑, Viszeral‑, Thorax- und Transplantationschirurgie der Uniklinik Köln

Kennzahlen der Weiterbehandlung der 39 Patient:innen im universitären High-Volume-Zentrum	
ø 44 Tage (6 bis 131 Tage) Krankenhausliegedauer	–
ø 22 Tage (1 bis 110 Tage) Liegedauer auf der Intensivstation	–
1 bis 43 Tage Beatmungsdauer	(2 Patient:innen: ECMO)
38 Patient:innen: Revisionseingriffe (Operation und/oder Intervention)	(27 Patient:innen: Revisionsoperationen, 37 Patient:innen: endoskopische interventionelle Revisionen, 14 Patient:innen: Kombination aus Operation und Intervention)
115 Operationen	(17 Patient:innen: mehrfach operiert, jeweils 3 bis 11 Revisionsoperationen)
243 diagnostische und/oder interventionelle ÖGDs	(171 EndoVAC-Wechsel)
148 CTs	(27 CT-gesteuerte Punktionen mit Drainageanlagen)

Nach der Verlegung in unser Zentrum wurden bei 38 Patient:innen (97,4 %) Revisionseingriffe im Sinne einer Operation oder Intervention durchgeführt. So wurden 27 Patient:innen (69,2 %) operiert und 37 Patient:innen (94,8 %) interventionell endoskopisch behandelt. Dabei gab es eine Überschneidung von 14 Patient:innen, die eine Kombination aus Intervention und Operation erhielten.

Die endoskopischen Interventionen gliederten sich in EndoVAC-Therapien und Stentanlagen im Bereich des oberen Gastrointestinaltraktes. Es wurden während der Weiterbehandlung bei 37 Patient:innen 243 diagnostische und/oder interventionelle Ösophagoduodenoskopien (ÖGDs) durchgeführt. Bei einer einzelnen Person wurden bis zu 28 ÖGDs im stationären Aufenthalt durchgeführt. Im Rahmen der 243 ÖGDs erfolgten 171 EndoVAC-Wechsel. Eine einzelne Person erhielt bis zu 25 EndoVAC-Wechsel. Die jeweiligen EndoVAC-Therapien verliefen durchschnittlich über 30 Tage mit einer maximalen Therapiedauer von 105 Tagen.

Die Weiterbehandlung der extern aufgetretenen Komplikationen erforderte bei 27 Patient:innen kumulativ 115 Operationen. Drei Patient:innen wurden 10- bis 11-mal in der weiterbehandelnden Klinik operiert.

Dabei waren die häufigsten Operationen Resektionen (darunter 6 Diskontinuitätsösophagektomien), 10 Rekonstruktionen der gastrointestinalen Passage, 20 Defektdeckungen und Übernähungen sowie thorakale und abdominelle Explorationen mit Lavage und/oder Drainageanlagen, Portexplantationen und thorakale, abdominelle und epifasziale VAC-Versorgungen. Insgesamt benötigten 63,0 % der Patient:innen (17/27) mehrere Revisionseingriffe (Range: 3 bis 11 Operationen in der Uniklinik Köln). Während der Weiterbehandlung der Patient:innen wurden in unserer Klinik insgesamt 148 Computertomographien (CTs) durchgeführt. Es erfolgten 27 CT-gesteuerte Punktionen mit Drainagenanlage sowie 4 diagnostische und/oder interventionelle Angiographien (Tab. 4).

Im Rahmen des stationären Aufenthaltes verstarben nach Übernahme 5 der 39 Patient:innen, was sich in einer Mortalität von 12,8 % widerspiegelt. Zu den Todesursachen zählten Multiorganversagen bei Sepsis (*n* = 3), abdominelles Kompartment bei umfassender Mesenterialischämie sowie schwerer hypoxischer Hirnschaden nach kardiopulmonaler Reanimation bei pulsloser elektrischer Aktivität. Die Patient:innen verstarben binnen 61 Tagen nach extern erfolgtem komplikativem Primäreingriff, innerhalb von 6 bis 38 Tagen nach Zentrumsverlegung. Alle Patient:innen verstarben auf der Intensivstation. Insgesamt konnten 61,5 % der Patient:innen mit extern komplikativ verlaufenden Eingriffen wieder in die Häuslichkeit entlassen werden. Weitere 25,6 % der Patient:innen konnten nach Stabilisierung des Allgemeinzustandes heimatnah verlegt werden.

**Abb. 1:**
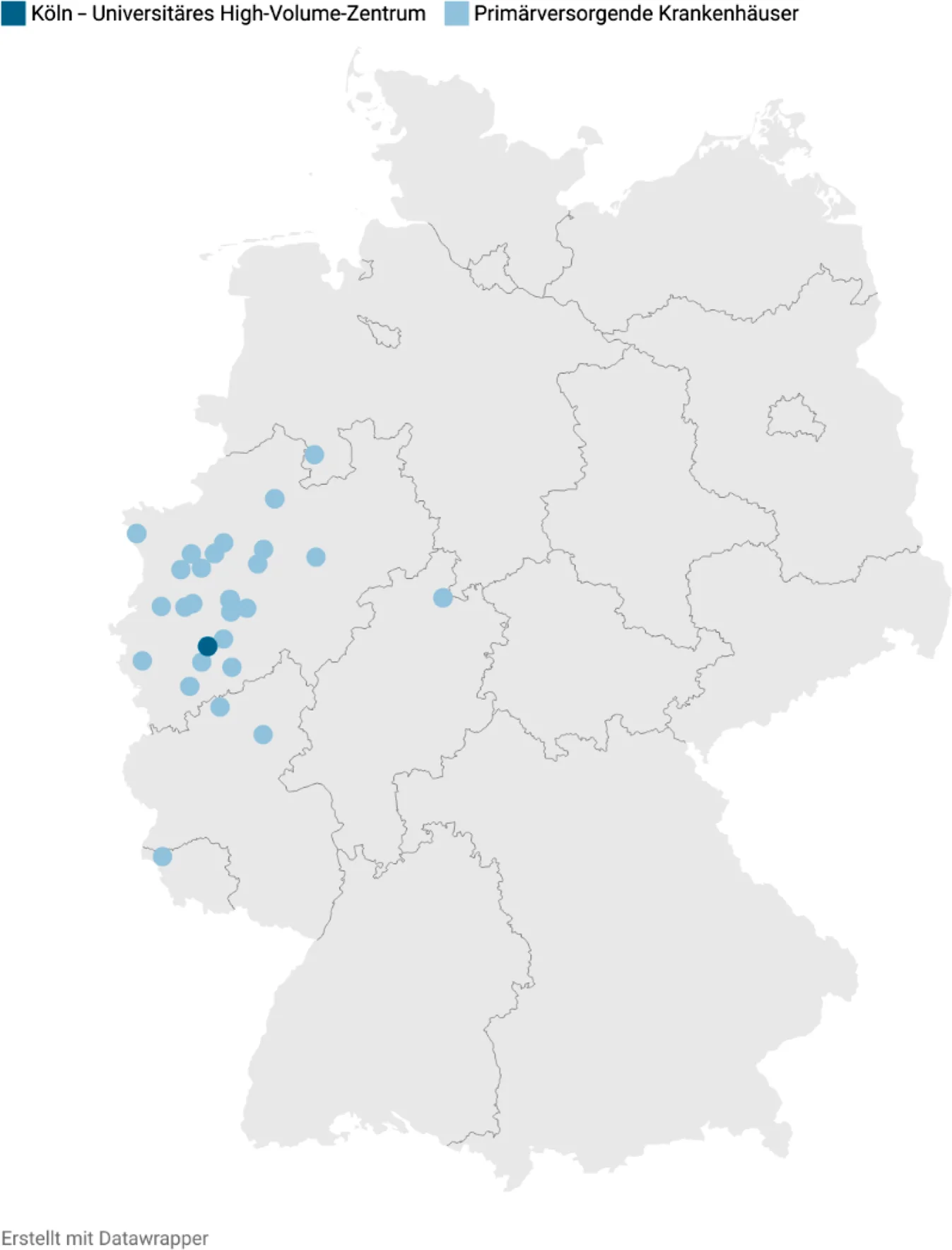
Geografische Übersicht: Lagebezug der primärversorgenden Krankenhäuser zum weiterbehandelnden universitären High-Volume-Zentrum (Uniklinik Köln). Deutschlandkarte erstellt mit Datawrapper

Die zuweisenden Krankenhäuser befanden sich in Nordrhein-Westfalen, Rheinland-Pfalz, Saarland, Hessen und Niedersachsen mit einer maximalen Distanz von ca. 210 km Luftlinie zur Uniklinik Köln (Abb. 1). Sie gehörten überwiegend zu Krankenhäusern der Grund- und Regelversorgung, aber auch zu Krankenhäusern der Schwerpunkt- und Maximalversorgung.

### Drei Fallbeispiele

Die Tab. 5 (s. Zusatzmaterial online) zeigt eine fallspezifische Aufarbeitung der 39 Patient:innen.

#### Fall 1

**Abb. 2:**
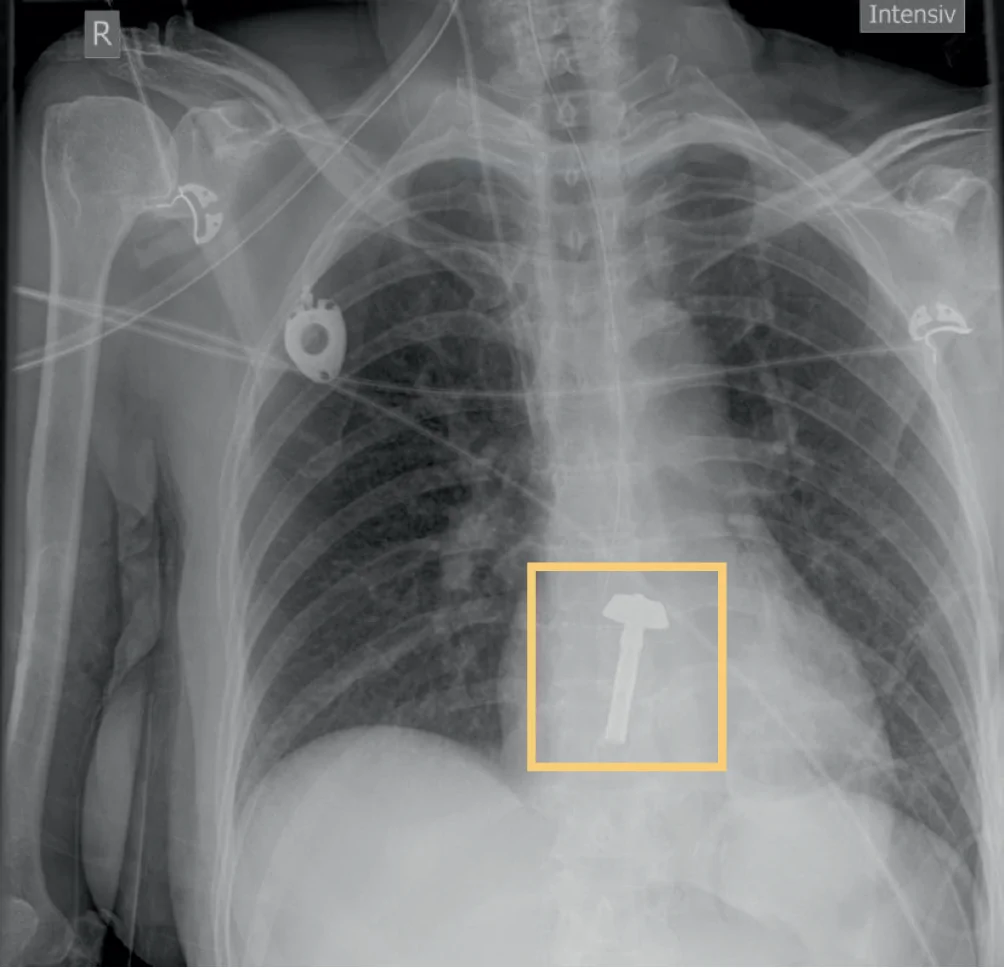
Röntgen-Thorax-Aufnahme in anterior-posterior Projektion im Liegen. Das Röntgenbild ist auf der Intensivstation direkt nach der Zentrumsverlegung entstanden. Extern erfolgte der Abbruch einer Gastrektomie bei technischer Inoperabilität mit vorzeitiger Beendigung der Operation ohne Wiederherstellung der gastrointestinalen Passage. Zu sehen ist der eingenähte, extern in-situ belassene Staplerkopf für die zuvor geplante Ösophagojejunostomie (vgl. Markierungskasten). (Quelle: UKK)

Eine 57-jährige Patientin mit einem Adenokarzinom des Magens mit Siegelringzellkomponente (ypT4a, N0 i + , L0, V0, Pn0, R1 [ösophageal, duodenal], pM1 [PER], G3), Z. n. neoadjuvanter Chemotherapie, erhielt eine elektive offene Gastrektomie in einem Krankenhaus der Grundversorgungsstufe. Intraoperativ wurde mittels Schnellschnitt ösophageal und duodenal eine inkomplette Resektion festgestellt. Während der Wartezeit auf die Begutachtung des Schnellschnitts wurde die Operation fortgeführt und der Zirkularstaplerkopf eingenäht. Aufgrund des positiven Schnellschnittes wurde die Operation ohne Wiederherstellung der gastrointestinalen Passage abgebrochen, der Staplerkopf verblieb im Operationssitus, und es erfolgte der Bauchdeckenverschluss (Abb. 2). Die Patientin wurde am Operationstag wach, ansprechbar und extubiert auf unsere operative Intensivstation übernommen. Am Folgetag erhielt die Patientin eine Relaparotomie mit Nachresektion mit Schaffung einer R0-Situation im Schnellschnitt sowie eine transhiatale, subtotale Ösophagusresektion mit Roux-Y-Rekonstruktion. Am 18. postoperativen Tag erfolgte bei Nachweis einer Portinfektion die Portexplantation. Bei Bridenileus wurde im weiteren Verlauf eine erneute Relaparotomie mit Adhäsiolyse durchgeführt. Die Patientin konnte schließlich nach 47 Tagen, davon 2 Tage in intensivmedizinischer Behandlung, wieder in die Häuslichkeit entlassen werden. Im Rahmen des stationären Aufenthaltes erhielt die Patientin eine ÖGD und eine CT-Diagnostik.

#### Fall 2

**Abb. 3:**
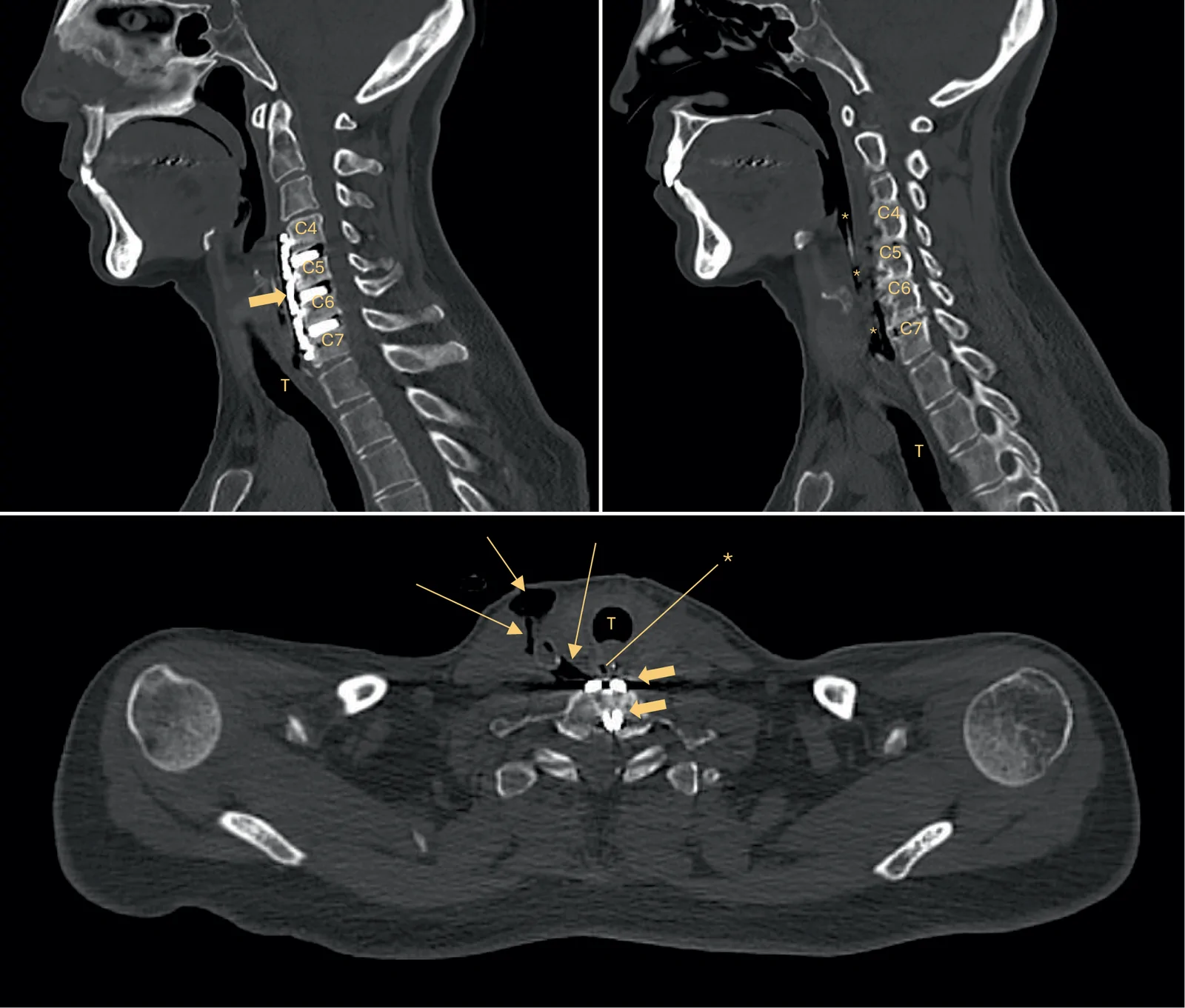
Zervikale CT-Aufnahmen in sagittaler (*oben*) und axialer (*unten*) Ebene. Zu sehen ist eine zervikale Ösophagusperforation mit zervikalem Lufteintritt durch eine Plattenosteosynthese der Halswirbelkörper C4–C7. Markierungen: *Sternchen* ≙ Ösophaguslumen; *schmale Pfeile* ≙ Luftperlen; *dicke Pfeile* ≙ Fremdmaterial der Plattenosteosynthese; T ≙ Trachea. (Quelle: UKK)

Eine 53-jährige Patientin erhielt bei einem Bandscheibenvorfall eine anteriore zervikale Diskektomie mit Fusion der Halswirbelsäule von C4–C7. Intraoperativ kam es zu einer Ösophagusperforation mit zervikaler Abszessbildung im Verlauf (Abb. 3). Die Patientin wurde extern endoskopisch mit einer VAC-Therapie versorgt und erhielt 3 Operationen zur zervikalen Abszesssanierung. Bei persistierendem Halsabszess mit ösophagokutaner Fistel erfolgte nach 33 Tagen nach Primäreingriff die Zuverlegung an unser Zentrum. Die Patientin wurde zunächst unfallchirurgisch mittels dorsaler Spondylodese C4–C7 versorgt und erhielt im weiteren Verlauf eine ventrale zervikale Metallentfernung und einen Bandscheibenersatz C6/C7 mittels allogenen Knochens. In der gleichen Operation erfolgte nach intraoperativer Endoskopie die Übernähung des Ösophagus und Hypopharynx, eine Defektdeckung mit einem gestielten rechtsseitigen Musculus-sternocleidomastoideus-Lappen. Im stationären Aufenthalt kam es zu einer kutanen Wundheilungsstörung, welche mit einer VAC-Therapie versorgt wurde. Die Patientin erhielt im weiterbehandelnden Zentrum kumulativ 6 Operationen, 4 ÖGDs und 4 CTs und konnte schließlich in die Häuslichkeit entlassen werden.

#### Fall 3

**Abb. 4:**
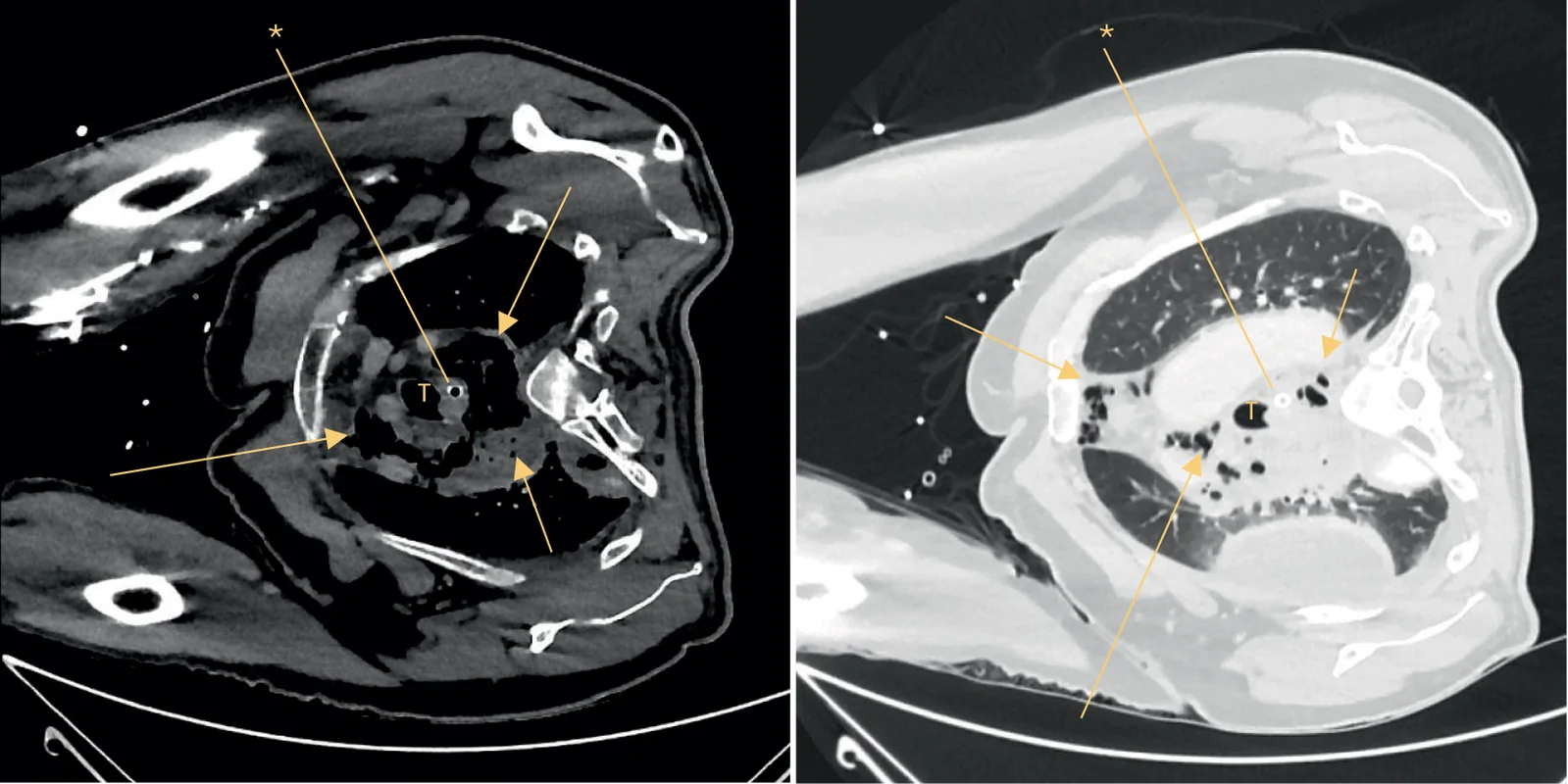
Thorakale CT-Aufnahmen in Seitenlage in axialer Ebene. Zu sehen sind ausgeprägte mediastinale Lufteinschlüsse bei Ösophagusperforation mit Mediastinitis nach endoskopischer Schwellenspaltung bei Zenker-Divertikel. Markierungen: *Sternchen* ≙ Ösophaguslumen; *schmale Pfeile* ≙ Luftperlen im Mediastinum; T ≙ Trachea. (Quelle: UKK)

Bei einem 66-jährigen Patienten kam es im Rahmen einer endoskopischen Schwellenspaltung mittels Laser bei Zenker-Divertikel zu einer Perforation des Ösophagus mit Entwicklung einer Mediastinitis (Abb. 4). Der Patient wurde nach Entdeckung der Perforation 7 Tage nach dem Primäreingriff ohne vorherige externe Revision an unser Zentrum auf die Intensivstation verlegt. Am Aufnahmetag erhielt der Patient eine CT-gesteuerte Drainagenanlage in den thorakalen Verhalt. Am Folgetag erfolgte die operative Versorgung mit Abszessentlastung und Übernähung der Ösophagusperforation in Kombination mit einer endoskopischen und zervikalen VAC-Therapie. Nach 74 Tagen konnte der Patient wieder nach Hause entlassen werden. Im Rahmen des Krankenhausaufenthaltes wurde der Patient 20 Tage intensivmedizinisch behandelt und erhielt insgesamt 10 Operationen und 12 ÖGDs mit einer EndoVAC-Therapie über 64 Tage.

Zusammenfassend lässt sich schlussfolgern, dass die Weiterbehandlung von extern komplikativ verlaufenden Eingriffen einer Vielzahl an Ressourcen (Tab. 4 und 5) bedarf. Dies zeigt sich insbesondere an den erforderlichen Kapazitäten für eine komplexe operative Versorgung und intensivmedizinische Betreuung mit kreislaufunterstützenden Maßnahmen sowie an der uneingeschränkten Verfügbarkeit von Diagnostik wie beispielsweise CTs und ÖGDs. Deutlich wird, dass eine hohe fachliche Expertise und eine intensive Zusammenarbeit verschiedener Fachdisziplinen von Bedeutung sind.

## Diskussion

Die Ergebnisse der vorliegenden Studie zeigen, dass sich die Versorgungsstrukturen in den erstbehandelnden Krankenhäusern beim Auftreten von Komplikationen unterscheiden. So erfolgte bei ca. zwei Dritteln der Patient:innen mit extern komplikativ verlaufenden Eingriffen innerhalb der ersten 30 Tage nach Auftreten der Komplikation eine Zentrumsverlegung und in einem Drittel der Fälle nach 30 Tagen. Nur 15 der 39 Patient:innen wurden direkt an das Zentrum verlegt ohne extern erfolgte interventionelle oder operative Revision. Die unterschiedlichen Zeitpunkte der Kontaktaufnahme mit einem Zentrum und der Zentrumsverlegung spiegeln wider, dass es keine übergreifenden fachlichen oder gesetzlichen Richtlinien für einen Zentrumswechsel beim Auftreten von operativen Komplikationen gibt. Auch die S3-Leitlinie „Perioperatives Management bei Gastrointestinalen Tumoren (POMGAT)“ definiert keine derartigen Handlungsempfehlen [9]. Das Fehlen einheitlicher Richtlinien verdeutlicht die hohe Komplexität von Komplikationen bei bereits anspruchsvollen Primäreingriffen. Das Management dieser Komplikationen erfordert somit eine interdisziplinäre Fachexpertise und bedarf individueller Einzelfallentscheidungen. Bei einigen Patient:innen erfolgten extern multiple Revisionen ohne Kontrolle der Komplikationen, sodass eine Zentrumsverlegung im Verlauf initiiert wurde. Dies lässt schlussfolgern, dass bei Komplikationen insbesondere von komplexen Operationen unmittelbar eine Kontaktaufnahme mit einem Zentrum erfolgen sollte. Die Intensivpflichtigkeit von 71,8 % und Beatmungspflichtigkeit von 25,6 % der Patient:innen zum Zeitpunkt der Verlegung legt den kritisch kranken Zustand dieser Patient:innen dar; 38 der 39 Patient:innen wurden in unserem Zentrum interventionell und/oder operativ versorgt. Die Mehrzahl der Patient:innen benötigte multiple Revisionseingriffe in domo. Die Aufführung der kumulativ erfolgten Operationen (*n* = 115 von insgesamt 5439 in den Jahren 2023 und 2024 in unserer Klinik durchgeführten Operationen ≙ 2,1 %), ÖGDs (*n* = 243 von insgesamt 1617 in den Jahren 2023 und 2024 durchgeführten ÖGDs ≙ 15 %) und CTs (*n* = 143) verdeutlicht den hohen Bedarf an personellen, apparativen und logistischen Ressourcen.

### Mindestmengen in der modernen Medizin

Die erste Pionierarbeit von Luft et al. zum Effekt von Leistungsmengen auf die Versorgungsqualität erschien 1979 im *New England Journal of Medicine* [10]. Luft et al. untersuchten den Zusammenhang von Mortalität und Fallzahlen für 12 Operationen unterschiedlicher Komplexität aus verschiedenen chirurgischen Fachbereichen. Die Autoren fanden eine Korrelation von höheren Fallzahlen und niedrigeren Mortalitätsraten insbesondere bei komplexen Operationen. Wachsendes internationales und nationales Interesse erhielt die Thematik der Mindestmengen im Gesundheitswesen jedoch erst in den 1990er-Jahren. Ließ sich zu Beginn in Deutschland bei unzureichender Studienlage und -qualität noch kein eindeutiger Trend des Effekts von Mindestmengen auf die Versorgungsqualität ablesen [11], so zeigte eine deutsche Studie im Jahr 2017 unter anderem eine signifikant geringere Sterblichkeit bei Eingriffen am Ösophagus in Krankenhäusern, die die Mindestmengen erreichen [12]. Dieser Effekt wurde auch für weitere viszeralchirurgische Operationen wie Pankreasresektionen und Nierentransplantationen, aber auch für den totalen endoprothetischen Knieersatz dargelegt [12, 13]. Der direkte Effekt von Volumen (höhere Fallzahlen) auf Outcome (geringere Sterblichkeit) in der medizinischen Versorgung wurde auch in einer deutschen Metaanalyse von 2021 gefunden [14].

### Internationaler Vergleich

Betrachtet man die Leistungszentralisierung und Implementierung von Mindestmengen im Bereich des oberen Gastrointestinaltraktes europaweit, so fällt auf, dass nur in 4 weiteren Ländern offizielle Mindestmengen für komplexe Operationen am Ösophagus/Ösophagektomien bestehen (Österreich: 10 Eingriffe, Schweiz: 12 Eingriffe, Belgien: 20 Eingriffe, Niederlande: 20 Eingriffe) [15]. In den Niederlanden wurde 2011 die seit 2006 bestehende Mindestmenge von 10 komplexen Eingriffen am Ösophagus auf 20 Eingriffe erhöht [16]. Seit 2011 besteht ebenfalls durch die Etablierung des „Dutch Upper Gastrointestinal Cancer Audit“ (DUCA) ein nationales Registernetzwerk, das eine präzise Erfassung aller Patient:innen, die wegen Tumoren des Ösophagus und Magens operiert werden, gewährleistet [17]. Niederländische Studien zeigten einen positiven Zusammenhang zwischen gesteigerten jährlichen Fallzahlen und verbessertem Outcome hinsichtlich der Mortalität, des Auftretens von Anastomoseninsuffizienzen, der Qualität der lokalen Lymphadenektomien sowie des 6‑Monats- und 2‑Jahres-Überlebens [16–18]. Es zeichnete sich zudem ein maximales Outcome-Plateau bei der Durchführung von 40 bis 60 Ösophagektomien pro Jahr pro Krankenhaus ab [19].

### Kritik an der Mm-R

Trotz dieser positiven Resultate wird die Mindestmengenregelung oft kritisiert. Nachteile der Mindestmengenregelung könnten ein schlechterer Zugang von Patient:innen zu komplexeren Operationen insbesondere in peripheren Regionen sein. Zudem kann das Nichterreichen einer Mindestmenge die Standortattraktivität eines Krankenhauses negativ beeinflussen und Auswirkungen auf die ärztliche Weiterbildung und Personalgewinnung haben [20]. Dieser Kritik ist entgegenzubringen, dass sich der Mindestmengenkatalog in Deutschland nach aktuellem Stand auf lediglich 14 Eingriffs‑/Behandlungsarten bezieht [5, 7]. Ein möglicher Lösungsansatz für eine qualitative Ausbildung und einen gesicherten Zugang zu diesen medizinischen Leistungen können Kooperationen zwischen verschiedenen Krankenhäusern sein.

### Einordnung der Studie, Limitationen und Ausblick

In Einklang mit unserer Literaturrecherche verdeutlicht das systematische Review von Scharfe et al. [21] die (bis auf wenige genannte Ausnahmen) persistierend unzureichende internationale und nationale Studienlage zum Effekt von Mindestmengen in der Medizin auf die Versorgungsqualität. Die meisten Studien basieren auf einem retrospektiven Studiendesign und bewerten Informationen aus Datenbanken wie der „Diagnosis Related Groups“(DRG)-Statistik. Konkrete Kontexte der Komplikationen und Versorgungsstrategien lassen sich so oftmals nur unzureichend rekonstruieren. Positiv hervorzuheben sind somit das prospektive Design der hier vorliegenden Studie sowie die vollständige Nachbeobachtung aller Patient:innen bis zum Zeitpunkt der Entlassung, heimatnahen Rückverlegung oder des Exitus letalis über insgesamt 28 Monate.

Noch seltener als der Effekt von Mindestmengen ist die Versorgung nach externen Eingriffskomplikationen erforscht. Eine deutsche Studie von Götte et al. beschäftigte sich beispielweise mit Komplikationen nach uroonkologischen Primäreingriffen, welche während der Anschlussheilbehandlung aufgetreten sind und in eine Weiterbehandlung in einer Akutklinik mündeten [22]. Die dargelegte Fallserienaufarbeitung zum Management von Komplikationen im oberen Gastrointestinaltrakt nach externen Primäreingriffen hat somit ein gewisses Alleinstellungsmerkmal. Mögliche Limitationen der Studie sind der deskriptive Charakter sowie die gebündelte Betrachtung von Komplikationen, die durch komplexe Operationen am oberen Gastrointestinaltrakt entstanden sind, mit Komplikationen, die durch diagnostische Verfahren (ÖGD, TEE) oder Operationen anderer Fachbereiche (Orthopädie, Thoraxchirurgie) hervorgerufen wurden und wahrscheinlich nicht durch das Erreichen von Mindestmengen beeinflusst sind. Dennoch ist die übergreifende Auswertung aller extern aufgetretenen Komplikationen im Bereich des oberen Gastrointestinaltraktes sinnvoll, um die erbrachten und erforderlichen Ressourcen zur Behandlung solch komplexer Fälle darzulegen. Es liegt nahe, dass die Zentralisierung stationärer Versorgungsstrukturen und die Einführung von Mindestmengenregelungen nicht nur die Primärversorgung, sondern auch die Versorgung von Patient:innen im Falle von Komplikationen beeinflussen. Es bedarf weiterer, insbesondere prospektiver Studien, um den Effekt von Mindestmengen auf die Versorgungsqualität zu untersuchen. Zudem sind weitere Studien sinnvoll, die sich mit dem Auftreten und der Behandlung von komplexen Komplikationen befassen, um zum einen die Patientensicherheit zu erhöhen und zum anderen die Gesundheitsökonomik in Bezug auf personelle, apparative und logistische Ressourcen gezielt zu verbessern.

## Schlussfolgerung

Insgesamt zeigte sich, dass die Weiterbehandlung von Patient:innen mit extern komplikativ verlaufenden Operationen und Behandlungen einen hohen Bedarf an personellen, logistischen und medizinischen Ressourcen aufwies. Es erforderte sowohl aufgrund der meist bestehenden Intensivpflichtigkeit als auch aufgrund der meist erforderlichen komplexen thorakoabdominellen Revisionseingriffe ein interdisziplinäres Management. So stellen Revisionsoperationen im oberen Gastrointestinaltrakt bei auswärtigen Ersteingriffen auch in einem spezialisierten High-Volume-Zentrum eine erhebliche interdisziplinäre Herausforderung dar.

## Fazit für die Praxis

Auch wenn Operationen stets ein generelles Komplikationsrisiko haben, konnten durch die vorliegende Studie folgende Schlussfolgerungen gefasst werden:

- Optimierung von präoperativer Diagnostik zur Selektion von Patient:innen mit einem erhöhten Risikoprofil. Zuweisung dieser Patient:innen an spezialisierte Zentren zur Verbesserung der Patientensicherheit und Versorgungsqualität,
- frühzeitige Kontaktaufnahme der erstversorgenden Krankenhäuser mit spezialisierten Zentren bei Auftreten von Komplikationen,
- Vermeiden von chirurgischen Revisionen durch die erstversorgenden Krankenhäuser.

## Supplementary Information

Zusätzliche Tab. 5: Fallserienaufarbeitung

## Data Availability

Die erhobenen Datensätze können auf begründete Anfrage in anonymisierter Form beim korrespondierenden Autor angefordert werden. Die Daten befinden sich auf einem Datenspeicher der Klinik und Poliklinik für Allgemein‑, Viszeral‑, Thorax- und Transplantationschirurgie des Universitätsklinikums Köln (Universität zu Köln, Medizinische Fakultät).
