# Supplementary material for: Management of complications of the upper gastrointestinal tract following external primary procedures: Case series on complex clinical courses and continuation of treatment at a high-volume center
Source: Chirurgie (Heidelb). 2026 Aug 10;97(11):901–10. [Article in German] doi: 10.1007/s00104-026-02553-7 (PMC13687252; doi:10.1007/s00104-026-02553-7)
Supplement: Supplementary file 1 [file 104_2026_2553_MOESM1_ESM.docx]

Tabelle 5: Fallserien-Aufarbeitung
